# Editorial: Lessons learned from the Rehabilitation International World Congress—moving societies

**DOI:** 10.3389/fresc.2022.1040013

**Published:** 2022-11-25

**Authors:** Reuben Escorpizo

**Affiliations:** Department of Rehabilitation and Movement Science, The University of Vermont, Burlington VT United States

**Keywords:** rehabilitation, health systems, disability, functioning, ICF, work

**Editorial on the Research Topic** Lessons learned from the Rehabilitation International World Congress—moving societies By Escorpizo R. (2022) Front. Rehabilit. Sci. 3: 1040013. doi: 10.3389/fresc.2022.1040013.

With the rise of chronic health conditions and comorbid conditions, if there is one area in medicine and health that is more critical now than in the last decades, it is the field of rehabilitation. Rehabilitation has been defined by the World Health Organization (WHO) as “a set of interventions designed to optimize functioning and reduce disability in individuals with health conditions in interaction with their environment” (1). According to the United Nations, the current world population stands at almost 8 billion (2), of which about 30% need rehabilitation service (1). Yet, access to rehabilitation remains limited for those who need it the most and this disparity is more profound in low and middle-income countries (1). As an organization, Rehabilitation International (RI) has been a strong advocate for advocating for the rights of people with disability and for promoting their inclusion in society (3). RI's core mission has been in support of the United Nations Convention on the Rights of Persons with Disabilities (CRPD) (4), which provides a universal platform to learn from and develop strategies for people with disability. A biopsychosocial framework like the International Classification of Functioning, Disability and Health (ICF) (5) proves useful in examining the effects of disability on a person and what possible interventions to promote their participation in the context of their environment. Rehabilitation and the ICF make the ultimate case for strengthening health systems.

In September 2021, RI organized its World Congress, showcasing and celebrating the work of disability researchers, stakeholders, and advocates with the theme “Moving Societies”. The theme is relevant to the aim of rehabilitation being a societal strategy and strengthening the health systems to have a healthy population (6). This research topic brings forth a collection of papers from RI's World Congress that reflects on the importance of rehabilitation and identifying the rehabilitation needs of people and the creative and innovative thinking, and diverse approaches to addressing those needs. RI promotes empowerment, giving access to and being inclusive of people regardless of disability and background. Based on the RI World Congress, this research topic highlights the value of rehabilitation in various areas such as cancer Désiron et al. (2022), brain injury Thøgersen et al. (2022), Parkinson's disease Tonnesen et al. (2022), physical activity and sports Stolz et al. (2022), occupational rehabilitation Johansen et al. (2022) and work organization Mulders et al. (2022), weight loss program Jessen-Winge et al. (2022), peer counseling Jordan (2022), and low vision rehabilitation Øien (2022). Finally, an article from the research topic proposes strategies to further develop the rehabilitation research Aadal et al. (2022).

Long-term health conditions such as brain injury and Parkinson's disease are associated with a significant burden on people and their families and caregivers, and rehabilitation goals are crucial to recovery. Thøgersen and colleagues tested a psychological-cognitive approach to capture a holistic perspective of a person with a moderate-to-severe acquired brain injury Thøgersen et al. (2022). The approach highlighted the need for self-awareness and guiding person-centered goals as part of a holistic rehabilitation program. According to Tonnesen and colleagues, goal setting is just as important as interventions. Persons with disability are the primary drivers of their goals in rehabilitation, and developing functioning goals requires time within a continuum of care Tonnesen et al. (2022).

Employment is an important outcome in rehabilitation because it illustrates a person's productivity and well-being at the individual and societal levels. This research topic highlights the work of Désiron and colleagues who argue that healthcare professionals are in a position to facilitate a return to work for people with cancer and must be able to identify the barriers and facilitators for a successful return to work and toward a shared goal Désiron et al. (2022). Johansen and colleagues outlined a project where the ICF served as an assessment tool to facilitate communication between clinicians, the patient, and job center professionals- a multi-stakeholder partnership found to affect successful work outcomes. While the ICF provided a good framework, they found it to be challenging in terms of an increase in the time burden with its administration Johansen et al. (2022). A practical perspective was offered by Mulders and colleagues with their concept of the “Inclusive Work Redesign” Mulders et al. (2022), which proposes a unique insight into how work is organized and puts people with disabilities in the mainstream, and is intended to help sustain the return to work for people with disabilities amidst current challenges in the labor force market and social security.

Rehabilitation encompasses multiple areas identified to need crucial attention and sensory loss such as vision loss is no exception. Øien conducted a comprehensive action research project highlighting the importance of the environment which overlaps with the biopsychosocial aspects of vision loss, particularly about the person-environment relationship. In this case, addressing the environment and lighting will enable people with vision loss to participate in their environments Øien (2022). Stolz and colleagues expressed the value of using the ICF framework in their work investigating the “Sportcoach” approach which mitigates work disability by way of participation in sports activities and engaging in a sports club, another holistic look at a way to innovate the delivery of rehabilitation Stolz et al. (2022).

Jessen-Winge and colleagues tackled the issue of obesity by developing strategies for effective rehabilitation programs. The authors found that awareness, activity patterns, pacing, social relationships, and balancing life are key elements of rehabilitation in the context of sensible weight loss Jessen-Winge et al. (2022). Rehabilitation programs will benefit from effective peer counseling- a process undertaken by and for people with disabilities. Jordan discussed the role that peer counseling can play in empowering people with disabilities across different kinds of health conditions Jordan (2022). Finally, Aadal and colleagues have reaffirmed the need to ground rehabilitation research in biopsychosocial principles and adhere to its cross-setting and multidisciplinary nature Aadal et al. (2022). Their work revealed the important role of service and end users, the use of the ICF, the continuum, and the ultimate steps of dissemination and implementation of findings.

Rehabilitation is characterized by a multidisciplinary approach and tackles the complex nature of physical, mental, cognitive, and multi-aspect disability in light of health conditions or diseases. Rehabilitation uses various platforms in research, education, policymaking, and practice to make a difference in people's lives by improving their functioning in their daily lives alongside their resiliency and prevention of further disabilities so they can accomplish their full potential. These characteristics make rehabilitation a keystone health strategy of society.

## References

[1] World Health Organization. Rehabilitation. [cited 2022 Sep 6]. Available from: https://www.who.int/news-room/fact-sheets/detail/rehabilitation

[2] World Population Dashboard. United Nations Population Fund. [cited 2022 Sep 6]. Available from: https://www.unfpa.org/data/world-population-dashboard

[3] Rehabilitation International. RI Global. [cited 2022 Sep 6]. Available from: https://www.riglobal.org/

[4] Convention on the Rights of Persons with Disabilities (CRPD) | United Nations Enable. [cited 2022 Sep 6]. Available from: https://www.un.org/development/desa/disabilities/convention-on-the-rights-of-persons-with-disabilities.html

[5] World Health Organization. International classification of functioning, disability and health (ICF). Geneva, Switzerland: World Health Organization (2001). p. 1–303.

[6] Stucki G Bickenbach J Gutenbrunner C Melvin J. Rehabilitation: the health strategy of the 21st century. J Rehabil Med. (2018) 50(4):309–16. 10.2340/16501977-2200 28140419

